# A Qualitative Descriptive Exploration of Stakeholder Perceptions of a National Ear, Nose, and Throat Emergency Course

**DOI:** 10.7759/cureus.97227

**Published:** 2025-11-19

**Authors:** Jemma Butler, Sharifah S Badrol, Jacquline Chan, Hannah R Lancer, Emma C Richards, Qabirul Abdullah, Emma Watts

**Affiliations:** 1 Otolaryngology, Worcestershire Acute Hospitals NHS Trust, Worcester, GBR; 2 Otolaryngology - Head and Neck Surgery, University Hospitals Coventry and Warwickshire, Coventry, GBR; 3 Otolaryngology, Birmingham Children's Hospital, Birmingham, GBR; 4 Otolaryngology - Head and Neck Surgery, University Hospitals Birmingham NHS Foundation Trust, Birmingham, GBR; 5 Centre for Medical Education, University of Dundee School of Medicine, Dundee, GBR

**Keywords:** blended learning approach, clinical competence, continuing medical education (cme), otolaryngology education, program evaluation, simulation training

## Abstract

Background: In the United Kingdom (UK), resident doctors frequently rotate through specialties, and many begin ear, nose, and throat (ENT) placements with limited prior experience, highlighting the importance of structured induction. The West Midlands ENT Emergency Course (WMEEC) is a national program combining lectures and practical skills stations to prepare new ENT doctors to manage common emergencies safely.

Objectives: This study aims to evaluate the WMEEC following a decline in faculty and delegate attendance by exploring the perspectives of key stakeholders involved in the December 2023 course.

Design: A qualitative descriptive study employing semi-structured focus groups and interviews, with structured processes for recruitment, data collection, and reflexive thematic analysis. The interview guide and analysis were informed by experiential learning and motivation theory. Ethical approval was granted by the University of Dundee and a local hospital trust, with informed consent obtained from all participants.

Setting: The WMEEC, delivered in the West Midlands, UK, consists of didactic lectures and hands-on practical skills stations.

Participants: Twenty-five stakeholders were invited using purposive sampling. Following the exclusion of senior house officers (SHOs) with direct supervisory relationships to the researcher, 13 participated. Participants represented five stakeholder groups: ENT SHO delegates, ENT SHO non-delegates, ENT registrar faculty, ENT registrar non-faculty, and ENT consultants.

Results: Reflexive thematic analysis generated 54 codes across three key themes: pedagogical framework, facilitators and barriers to participation, and operational structure. Participants viewed the course as valuable, particularly for its practical sessions and concise lectures, and perceived improved confidence among SHOs following attendance. Faculty were motivated by altruism and a desire to support training, while SHOs were driven by skill development and career progression. Reported barriers included course cost and weekend scheduling.

Conclusions: This study provides stakeholder-informed insights into the strengths and limitations of the WMEEC. Participants suggested that incorporating blended or online learning may enhance accessibility and sustainability. While findings are specific to one region and course, they highlight the value of ongoing, stakeholder-driven evaluation in improving specialty induction programs.

## Introduction

Structured induction programmes play a crucial role in preparing trainees for new clinical environments and responsibilities [1]. When effectively implemented, they provide a safe, standardised environment to practise essential procedural and communication skills before commencing rotations [2,3]. In line with recommendations from the General Medical Council (GMC) and national surgical training frameworks [4], simulation-based induction has been widely adopted to enhance preparedness and patient safety across specialties [5].

In the United Kingdom (UK), resident doctors frequently rotate through various specialties, and many begin ear, nose, and throat (ENT) placements with little to no prior experience, as undergraduate exposure to ENT is often limited [6]. ENT-specific induction programmes remain inconsistent across the UK, with considerable variation in course structure; moreover, previous evaluations have largely reported quantitative outcomes (e.g., satisfaction and self-reported confidence), with few studies qualitatively exploring stakeholders’ perspectives and contextual barriers [7]. Although 90% of courses address seven core ENT skills and are mainly registrar-led, they are often brief, averaging just 4.6 hours [8]. Access is restricted by cost and geographical barriers, with course fees ranging from $50 to $264. Most are delivered in major teaching hospitals, meaning trainees based in smaller or rural centres often face additional travel and accommodation costs, which can make attendance challenging [8].

The West Midlands ENT Emergency Course (WMEEC) was established to address this gap, offering a comprehensive, one-day induction for resident doctors commencing ENT placements. The course combines interactive, case-based lectures in the morning with afternoon practical simulation stations covering core emergency procedures such as epistaxis control, tracheostomy care, and foreign-body removal. Designed and delivered by regional ENT registrars, the WMEEC aims to improve participants’ confidence, clinical preparedness, and indirectly patient safety through experiential, near-peer learning. Since its inception, the WMEEC has undergone minimal structural change and lacks a formal curriculum. Although historically popular, recent declines in attendance and faculty engagement prompted a formal evaluation.

This study aims to qualitatively explore the perceptions and experiences of key stakeholders involved in a national ENT emergency course, with the goal of understanding barriers to engagement and informing future educational design and policy. Specifically, it seeks to address the following research questions: (1) What do stakeholders perceive as the strengths and weaknesses of the WMEEC? (2) What practical, financial, and social factors influence faculty and delegate engagement with the course? (3) How do stakeholders perceive the relevance and delivery format of the course, and how might these insights inform future curriculum design?

## Materials and methods

A qualitative descriptive design was adopted, employing semi-structured focus groups and interviews to explore stakeholder perceptions and experiences of the WMEEC held on December 2, 2023. Ethical approval was granted by the University of Dundee and a local hospital trust.

A purposive sampling approach was used to capture a broad range of perspectives from five stakeholder groups within the WMEEC: ENT SHO delegates, ENT SHO non-delegates, ENT registrar faculty, ENT registrar non-faculty, and ENT consultants. Five individuals were invited from each group (n = 25). In total, 13 participants took part in the study, representing an overall response rate of 52%. The achieved sample comprised four ENT SHO delegates, three ENT SHO non-delegates, three ENT registrar faculty members, two ENT registrar non-faculty, and one ENT consultant. The corresponding response rates for each group were 80%, 60%, 60%, 40%, and 20%, respectively.

Three focus groups were conducted: Focus Group 1 (five faculty registrar participants), Focus Group 2 (four SHO non-delegate participants), and Focus Group 3 (two SHO delegate participants). In addition, two individual interviews were undertaken with the participants who were unable to attend group sessions due to clinical or on-call commitments. Due to lower response rates, individual interviews were conducted with the consultant and non-faculty registrar stakeholders instead of focus group discussions.

All sessions followed the same semi-structured question guide, and all transcripts - including those from individual interviews - were analysed together within a single reflexive thematic analysis framework to ensure consistency across stakeholder groups. The interview data were fully integrated with the focus group findings using the same coding process and theme development. Non-participation was primarily attributed to rota clashes, last-minute clinical duties, and annual leave. All participants met the inclusion criteria (currently working in ENT and either attended or were eligible to attend the December WMEEC). Although only one consultant and one non-faculty registrar participated, these individuals provided rich, detailed insights that were fully integrated with the focus group data. Thematic saturation was demonstrated across the dataset, as no new ideas emerged during later analyses. Given the qualitative design, the intention was to capture a broad range of experiences rather than proportional representation of each stakeholder group.

The lead researcher, an ENT registrar and long-standing faculty member of the WMEEC, provided valuable contextual insight but also potential for bias. As a peer to some registrar participants and junior to consultant participants, the researcher maintained clear professional boundaries throughout recruitment and data collection to minimise any influence of existing working relationships. Participants were informed of the researcher’s dual role at the outset, and SHOs under the researcher’s direct supervision were excluded to avoid power-related bias. Other members of the research team had no formal teaching, supervisory, or organisational ties to the WMEEC or to the participants.

Semi-structured focus groups were used to explore both collective and individual experiences. The discussion guide (Appendix A) was designed by the research team, drawing on findings from a focused literature review and guided by the principles of qualitative interviewing outlined by Braun et al. [9,10]. Questions explored barriers to attendance, course structure, and impacts on confidence and career aspirations, with open-ended prompts encouraging narrative and reflective responses [11].

The discussion guide was piloted with two SHOs and two registrars who were not part of the main study. Focus groups (~45 minutes) were held within two weeks of the course, either in person or online, in familiar and private settings. Ethical standards were upheld throughout, with written informed consent obtained from all participants, who were reminded of confidentiality and their right to withdraw at any time. Discussions were audio-recorded with consent and supported by field notes and a reflective journal. Field notes were used to capture non-verbal cues and contextual details that enriched interpretation, while reflective notes promoted researcher awareness and reduced bias. These measures, together with real-time clarification of participants’ comments during focus groups, helped maintain rigour and enhance the credibility of the findings.

Data Analysis

All focus group and interview recordings were manually transcribed verbatim by the lead researcher, anonymised, and stored securely. No automated transcription software or external services were used. To ensure accuracy, the lead researcher re-listened to all recordings during transcription, and the blinded colleague who assisted with coding independently reviewed sample sections of each transcript against the audio.

Data were analysed using reflexive thematic analysis following Braun et al.’s six-step framework. This method was chosen for its flexibility and suitability for qualitative descriptive research, allowing interpretation of patterns in participants’ accounts without the theoretical constraints of grounded theory or mixed methods [12]. Transcripts were coded manually using Microsoft Word and Excel (Microsoft® Corp., Redmond, WA); no specialist qualitative analysis software was employed. Familiarisation involved repeated reading of transcripts to identify meaningful text segments, which were labelled with descriptive codes in Word. Codes were then collated and organised in Excel to enable comparison across participants and grouping into broader subthemes.

A preliminary codebook was developed collaboratively by the lead researcher and the second coder to document code definitions and promote consistency. Coding discrepancies were discussed and resolved, after which the final coding framework was applied to all transcripts.

**Figure 1:**
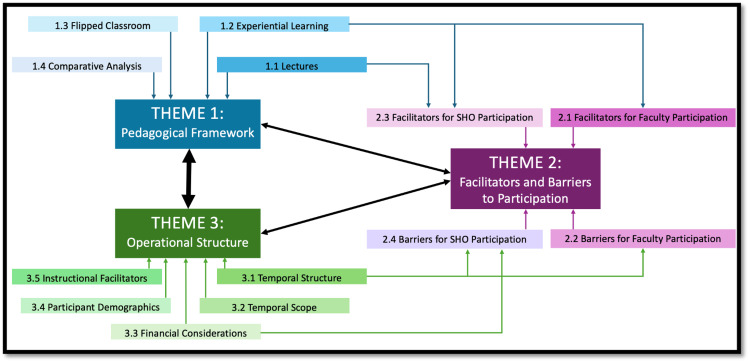
Thematic network diagram

Themes were constructed iteratively through comparison and refinement of related subthemes, guided by Braun et al.’s reflexive approach [9]. The process of theme generation and relationships between themes is illustrated in Figure 1. Appendix B shows the worked coding progression (from raw data extract to code, subtheme, and overarching theme), and an illustrative quote table supporting each theme is provided.

After completing the thematic analysis, a SWOT (strengths, weaknesses, opportunities, and threats) analysis was conducted by the research team to translate the qualitative findings into practical insights. The two researchers who coded the data reviewed each theme and subtheme collaboratively, mapping them to the SWOT categories of strengths, weaknesses, opportunities, and threats. This process was grounded entirely in the qualitative dataset, and consensus was reached through discussion to ensure consistency and transparency.

Rigour Measures

The analysis was carried out using a reflexive thematic approach, focusing on thoughtful interpretation rather than statistical agreement between coders. The lead researcher conducted the main coding while maintaining a reflective record to consider how their own background as a course faculty member might shape interpretation. A second researcher reviewed the developing codes and themes to offer another viewpoint and prompt reflection, rather than to measure reliability. Any differences were discussed to broaden understanding and improve the depth of the analysis.

Both surface meanings (what participants said directly) and underlying ideas (what their words suggested) were explored. Early coding focused on clear, descriptive points, while later analysis examined the beliefs, values, and experiences that shaped those views. This iterative process of reflection and refinement helped strengthen the coherence and connection of the main themes.

Rigour was supported through repeated reading of transcripts, reflection by the lead researcher after each stage, and discussion with the second coder. Themes were reviewed to ensure they were clear, coherent, and represented the range of perspectives across stakeholder groups. The lead researcher also maintained a reflective journal throughout the study to document assumptions, emotional responses, and methodological decisions. Regular peer discussions with an independent academic colleague not involved in the WMEEC were used to challenge interpretations and enhance transparency. Independent coding by a second researcher further strengthened reflexivity and analytical rigour.

## Results

Twenty-five stakeholders were invited, of which 13 agreed to participate and were categorised into five key stakeholder groups (Table 1).

**Table 1:** Summary of key stakeholder groups SHO: senior house officers

Key Stakeholders	No. of Participants	Participant Code(s)
Consultant	1	CONS 1
Non-faculty Registrars	1	REG 1
Faculty Registrars	5	REG 2, REG 3, REG 4, REG 5, REG 6
SHO Attendees	2	SHO 1, SHO 2
SHO Non-sttendees	4	SHO 3, SHO 4, SHO 5, SHO 6

Three primary themes emerged consistently (Table 2).

**Table 2:** Key themes and subthemes

Theme	Subtheme	Number of Codes
1. Pedagogical Framework	1.1 Lectures	3
	1.2 Experiential Learning	5
	1.3 Flipped Classroom	4
	1.4 Comparative Educational Analysis	3
2. Facilitators and Barriers to Participation	2.1 Facilitators for Faculty Participation	10
	2.2 Barriers for Faculty Participation	4
	2.3 Facilitators for SHO Participation	5
	2.4 Barriers for SHO Participation	6
3. Operational Structure	3.1 Temporal Structure	4
	3.2 Temporal Scope	3
	3.3 Financial Considerations	3
	3.4 Participant Demographics	2
	3.5 Instructional Facilitators	4

Theme 1: Pedagogical framework

This theme explored perceptions surrounding the structure and delivery of key course content alongside an analysis of suggestions for improvement (Table 3).

**Table 3:** Subthemes and codes related to the pedagogical framework

Theme	Subtheme	Code
1. Pedagogical Framework	1.1 Lectures	1.1.1 Content; 1.1.2 Structure; 1.1.3 Handouts
	1.2 Experiential Learning	1.2.1 Proformas; 1.2.2 Structure; 1.2.3 Evaluation of Existing Practical Sessions; 1.2.4 Recommendations for Additional Practical Sessions; 1.2.5 Formative Assessment
	1.3 Flipped Classroom	1.3.1 Positive Experiences on Flipped Classrooms; 1.3.2 Negative Perceptions on Flipped Classrooms
	1.4 Comparative Educational Analysis	1.4.1 Local Inductions; 1.4.2 Other ENT Emergency Courses; 1.4.3 Other Specialty Inductions

Lectures

Stakeholders valued the lectures for efficiently delivering core ENT knowledge, but noted concerns around retention and engagement. Time constraints were cited to explain the limited opportunities for interactive elements. Faculty highlighted the need for updating lecture content, while attendees appreciated that registrars introduced clinical relevance to basic knowledge content. Suggestions included pre-course handouts, although concerns around cognitive overload were noted: "I don’t think purely knowledge-based content is sufficient for induction" (SHO 3).

Experiential Learning

Practical stations were praised across all groups for their real-world applicability, skills development, and confidence-building impact on junior doctors. Faculty recommended improvements, including equipment lists, clear learning objectives for each station, and enhanced fidelity. Demonstration followed by practice and case discussions were valued, although concerns about judgment anxiety were raised: "Those are the key [practical skills]… if your SHO can do those things on call, you’re super happy as a registrar" (REG 1).

Flipped Classrooms

Pre-recorded lectures were supported for their accessibility and potential opportunity for post-course revision. Although faculty members felt that this would free up time, providing an opportunity for more practical sessions, they highlighted concerns about poor engagement prior to the course. SHOs echoed these concerns about lengthy recordings, which can lead to reduced attention. Live sessions were still valued for providing the opportunity to ask questions and enabling peer interaction: "If you have pre-recorded lectures, you’ve got the chance to do it perfectly. So you're going to give the most optimum version of that lecture" (REG 1). "If the pre-recorded lectures got too long, I’d probably just Google it… I would find the shortest video possible to get the information across quicker" (SHO 5).

Comparative Educational Analysis

Induction practices varied across departments and were often limited to administrative orientation without practical ENT training. The WMEEC was viewed by faculty as providing the most comprehensive induction program: "I think there’s more of a cultural change into doing stuff locally and trying to support the SHOs better" (REG 5).

Theme 2: Facilitators and barriers to participation

Prompted by a recent decline in faculty volunteers and SHO participants, this section explored motivations and barriers to course attendance (Table 4).

**Table 4:** Subthemes and codes related to facilitators and barriers to participation

Theme	Subtheme	Code
2. Facilitators and Barriers to Participation	2.1 Facilitators for Faculty Participation	2.1.1 Helping Others; 2.1.2 Enjoyment of Teaching; 2.1.3 Enhancing SHO Capabilities to Support ENT Registrars During On Calls; 2.1.4 Simplifying the Training of New SHOs; 2.1.5 Impact on SHO Performance; 2.1.6 Teacher Training Opportunities; 2.1.7 Payment of Facilitators; 2.1.8 Networking Opportunities; 2.1.9 Additional Benefits; 2.1.10 Mandatory Attendance for Registrars
	2.2 Barriers for Faculty Participation	2.2.1 Personal Reasons; 2.2.2 Teacher Training Opportunities; 2.2.3 Lack of Direct Benefit; 2.2.4 Course Timing
	2.3 Facilitators for SHO Participation	2.3.1 Acquiring ENT Knowledge; 2.3.2 Development of ENT Practical Skills; 2.3.3 Career Development; 2.3.4 Impact on SHO Confidence; 2.3.5 Mandatory Attendance for SHOs
	2.4 Barriers for SHO Participation	2.4.1 Personal Reasons; 2.4.2 Changing Attitudes to Training; 2.4.3 Course Timing; 2.4.4 Clinical Commitments; 2.4.5 Adequacy of Local Training; 2.4.6 Cost

Facilitators for Faculty Participation

Altruism was the primary motivator for faculty, with personal experience encouraging their involvement in teaching: "I personally went on the course… so I think it’s useful to help the next generation" (CONS 1).

Views on payment of faculty were divided; some supported financial incentives, others feared fee increases. Course-trained SHOs were perceived as more confident, capable, and beneficial to local services.

Barriers for Faculty Participation

Weekend personal and family commitments limited registrar availability, particularly among those who had fulfilled their mandatory teaching obligations for their own training progression. Some hesitated to teach SHOs from outside their deanery or felt it was time for newer colleagues to assume teaching roles.

Facilitators for SHO Participation

SHOs were primarily motivated by the opportunity to develop practical skills, with additional benefits including increased confidence, valuable networking experiences, and nurturing an interest in pursuing a future career in ENT. Portfolio-related incentives were suggested to encourage continued participation: "So if the course would give you some kind of points you could put on your CV, I think that would really get people through the door" (SHO 5).

Barriers for SHO Participation

Non-attending SHOs cited a lack of awareness or a pre-existing confidence in their practical skills. Some registrars believed SHOs were unwilling to sacrifice their time during weekends, and consultants questioned shifts in SHO motivation: "Back in my day, you wanted to be good at your job… Now I think this has shifted to just a job which pays the bills" (CONS 1).

Such presumptions were contradicted by attending SHOs, who valued weekend scheduling to avoid rota conflicts.

Theme 3: Operational structure

This final theme examined practical course considerations and suggestions for improvement (Table 5).

**Table 5:** Subthemes and codes related to operational structure

Theme	Subtheme	Code
3. Operational Structure	3.1 Temporal Structure	3.1.1 Attending Courses at Weekends; 3.1.2 Attending Courses on Normal Working Days; 3.1.3 Days in Lieu; 3.1.4 Clinical Commitments
	3.2 Temporal Scope	3.2.1 One-Day Course; 3.2.2 Two-Day Course; 3.2.3 Half-Day Course
	3.3 Financial Considerations	3.3.1 Perspectives on Current Course Costs; 3.3.2 Study Budget; 3.3.3 Self-Funding of Courses
	3.4 Participant Demographics	3.4.1 Prioritising Local SHOs; 3.4.2 Allied Healthcare Practitioners
	3.5 Instructional Facilitators	3.5.1 SHOs; 3.5.2 Registrars; 3.5.3 Consultants; 3.5.4 Formal Teacher Training

Temporal Structure

Registrars preferred weekend scheduling to avoid clinical disruption. However, SHOs without ENT aspirations were less motivated to attend outside of normal working hours. Some suggested taking time off for attendance if the local induction was insufficient, while others worried that this would affect clinical service provision. Time-off-in-lieu for faculty was proposed but not universally valued, with registrars noting: "[Attendees] are often more grateful… they recognise you’re doing it in your unpaid weekend time" (REG 1).

Temporal Scope

Stakeholders preferred the one-day format for efficiency and concentration: "If it’s all just in one day… then you can relax for the rest of the weekend" (SHO 2). Extending the course risked logistical complications, yet shortening it risked losing vital content.

Financial Considerations

The £65 course fee was seen as affordable, operating on a not-for-profit basis. Consultants advocated for deanery study budgets to cover fees: "I think it should come out of the study budget, because it’s helping you do your job" (CONS 1). Slow reimbursements deterred some applications. Financial barriers were acknowledged but not seen as major obstacles by most stakeholders.

Participant Demographics

The course primarily targeted local SHOs, though attendance was opened to external candidates if capacity allowed. Consultants noted external delegates might feel freer to engage: "(Non-local SHOs) might feel less embarrassed to ask questions because there’s no one to judge them… [they think] ‘I’m never going to meet these people again’" (CONS 1). Expansion to allied healthcare professionals was supported if feasible.

Instructional Facilitators

Faculty registrars, despite lacking formal teaching training, were considered ideal educators due to their approachability and frontline experience. Consultants were seen as better suited for lectures. Strengthening SHO-registrar relationships was mutually beneficial: "When you’re the SHO on call, you’re mostly going to be liaising with registrars. So it’s more important to develop that sort of interaction" (REG 3).

## Discussion

Reflexive thematic analysis identified three key themes: the pedagogical framework, participation factors, and operational structure. Stakeholders praised the course’s efficient delivery of lectures and skills stations, although concerns about knowledge retention prompted suggestions for improvement. Faculty engaged altruistically, while SHOs were motivated by practical skill acquisition. Weekend timing, logistics, and costs were moderate barriers to participation. Overall, the course was highly valued; however, opportunities exist to further enhance its delivery, accessibility, and sustainability.

This discussion interprets the findings through two complementary theoretical lenses: experiential learning theory, which explains how confidence and competence develop through practice and reflection, and motivation theory, which explores the intrinsic and extrinsic factors influencing engagement. Following the literature review, these frameworks guided question design and later informed interpretation. Thematic analysis was carried out inductively, with themes drawn directly from the data before being examined through these theoretical perspectives to explain patterns in learning, motivation, and engagement.

Researcher insider status was utilised to motivate engagement and provide understanding. All participants agreed that emergency ENT training is crucial; however, opinions diverged on its structure and delivery. Attendance improved delegate confidence, consistent with previous findings [13]. These findings reflect principles of experiential learning, where observing and practising skills in a supportive environment promotes self-efficacy and professional confidence through reflection and role modelling [14]. Simulation-based training built confidence through repetition, teamwork, and alignment with the development of a community of practice [15]. Motivational differences reflected Herzberg’s two-factor theory [16], where differing intrinsic and extrinsic factors shaped engagement. Faculty were primarily motivated by professional fulfilment and the desire to support trainee development, while SHOs balanced career progression with personal wellbeing and practical considerations, such as time and cost. Practical skill acquisition motivated all SHOs, with CPD accreditation providing an additional external incentive for participation.

Weekend scheduling improved accessibility, despite some stakeholders’ reservations. The preference for weekend delivery also reflects a broader shift in learning culture within postgraduate education, as trainees increasingly seek sustainable work-life integration and value protected personal time alongside professional learning commitments. Virtual learning was proposed to enhance accessibility and support adult self-directed learning. Virtual learning may also mitigate cognitive overload, as well as financial and scheduling barriers [17]. In-person simulation is likely to remain the standard due to limited evidence for the use of augmented reality in ENT education [18].

Suggestions for improvement

The COVID-19 pandemic highlighted the WMEEC's vulnerability to external challenges. Following the results of our thematic analysis, we performed a SWOT analysis to generate actionable insights for improvement. Strengths included the presence of experienced faculty and the use of simulation-based medical education. Weaknesses were noted in relation to course evaluation and the implementation of formative assessment. Opportunities lie in harnessing technological advancements and fostering faculty development, while the main threat identified was competition from other courses.

Lecture intensity formed a recurrent theme across all groups, with flipped classrooms discussed as a potential strategy to reduce cognitive load and enhance engagement. Participants expressed mixed views on this approach - while some valued the flexibility of pre-learning, others preferred interactive, in-person teaching. These perspectives highlight the importance of social interaction and experiential learning, where discussion and feedback support confidence and identity formation among SHOs. When effectively implemented, flipped learning can support the application of higher-order knowledge within the classroom and promote longer-term information retention [19]. Online practical skills delivery shows promise, with preliminary evidence suggesting its potential to reduce operative times and error rates [20]. However, concerns remain about replicating hands-on tactile experience and real-time feedback [21]. Simulation and VR offer potential for skills enhancement in the future but would require rigorous evaluation before implementation [22]. An open-source learning management system such as Moodle [23] can enhance accessibility by delivering pre-recorded modules designed according to the FAIR (findable, accessible, interoperable, and reusable) principles outlined by Harden et al., providing feedback, encouraging active engagement, supporting individualisation, and ensuring relevance [24]. These reusable learning objects support self-directed learning [25]; help mitigate cognitive overload; and offer efficient, flexible, and cost-effective mechanisms to deliver consistent, high-quality learning experiences [26]. Online asynchronous fora could further boost interactivity.

Informal assessment provides scope for self-reflection, demonstrates knowledge, and creates an environment for constructive feedback. Objective assessment methods such as multiple-choice questions (MCQs) could foster retrieval practice and gap identification [27]. Practical skill evaluation remains challenging due to debates over simulation validity [28]. Self-assessment and collaborative feedback are evidence-based pedagogies that could be introduced to the WMEEC to support knowledge transfer and encourage intrinsic motivation while supporting individual adaptability [29].

Robust evaluation is essential for sustainable education; however, SWOT analysis identified this as a significant weakness within the WMEEC [30]. Current WMEEC survey-based methods focus too narrowly on operational structure (Theme 3) and underutilise registrar perspectives, which are better suited to improving future course design. Ongoing iterative evaluation involving diverse stakeholders is needed. Likert scales can provide information on operational structure, while open-ended questions will be utilised for course improvements [31].

Competition from similar programs threatens enrolment numbers at the WMEEC and highlights the necessity for regular course review to maintain the relevance of WMEEC. Priorities should include online learning, formative assessment, and iterative evaluation to ensure sustainability.

Limitations

Purposeful sampling aimed to capture a range of stakeholder views; however, a 52% response rate risked non-response bias [32], and the inclusion of only one consultant restricted triangulation across senior stakeholder groups. While only one consultant and one non-faculty registrar participated, their contributions offered rich contextual insights, and no new themes emerged, suggesting adequate information power for this exploratory study. The non-faculty registrar’s perspective was influenced by their prior involvement in previous course iterations, which again introduced bias. The lead interviewer’s status risked positivity bias and potentially influenced the direction of questioning and authenticity of responses due to question-response reflexivity [33]. While focus groups and extended interviews provided rich data, they also introduced complexities that reduced the reliability of data interpretation [34]. Concerns around online interviews impacting interviewer-participant dynamics were outweighed by the value of secondary analysis of audiotaping to reduce data misinterpretations [35].

## Conclusions

This qualitative study provides novel insights into doctors’ experiences of the WMEEC, drawing on perspectives from a range of stakeholders. The course’s comprehensive practical content was highly valued; however, concerns were raised about logistical barriers, financial costs, and the sustainability of knowledge retention. Participants perceived improved confidence and preparedness following attendance at the WMEEC, which may contribute to smoother trainee transitions and, indirectly, to patient safety. Addressing barriers to access and reinforcing learning over time are essential to maximise the educational impact of such courses. By capturing diverse stakeholder views, this study offers a framework for evaluating and refining future ENT and specialty induction programmes. Sharing these findings within the wider ENT community of practice may help inform the development of sustainable training pathways that support professional growth.

## Appendices

Appendix A: Semi-structured focus group discussion (FGD) questions

Semi-Structured FGD 1: SHO Delegate

Thank you for agreeing to take part in this study looking at the perceptions of ENT SHO induction. This session will last approximately 30 minutes, and your responses will be anonymised.

Course Attendance

·       Did you attend the ENT Emergency Course? Why?

·       What aspects did you find most helpful? Why?

·       What would you change about the course? Why?

·       Did the ENT Emergency Course prepare you for clinical practice? Can you provide examples?

·       Do you think this course will help you beyond your immediate ENT placement (e.g., CPD points, surgical applications)?

·       Have you attended any other ENT emergency courses? Have you seen any other courses advertised?

Course Structure

·       Who were your main teachers? Would you prefer to be taught by consultants, registrars or someone else?

·       What did you think of the current structure and timing of the course? (i.e., morning lectures, afternoon practical sessions)?

·       How would you ideally like the course to be structured?

·       Are there any additional topics or sessions you wish the course had covered?

·       What was your opinion on the duration of the course?

·       How would you feel if we extended the course over two days? e.g., a full day of lectures and a full day of practical sessions

·       How would you feel if we shortened the course? e.g., pre-recorded morning lectures could be watched in your own time, and we could just provide the practical course. Would you watch them? Would you want them to be available after the course?

ENT Placement

·       How did you feel prior to starting your ENT placement?

·       How would you rate your knowledge and practical skills prior to starting in ENT?

·       Did you feel sufficiently prepared for starting your ENT placement?

·       Have you ever considered a career in ENT?

·       Did your career aspirations influence your decision to attend the course or vice versa?

Cost

·       Did you have to pay for the course?

·       Was this reimbursed through your study budget?

·       Do you think the price of the course was fair?

·       What would be the maximum amount of money you would be willing to spend on an induction course?

·       Do you think SHOs should pay for an induction to a specialty?

Timing

·       Did you have to attend the course on a scheduled off day? Did this influence your decision to attend the course?

·       How do you feel about participating in courses out of hours?

·       Were you granted time in lieu?

Inductions

·       What induction was provided by your local department?

·       What is your experience of induction in other specialties?

·       Was your local induction sufficient for commencing a placement in ENT?

Semi-Structured FGD 2: SHO Non-Delegate

Thank you for agreeing to take part in this study looking at the perceptions of ENT SHO induction. This session will last approximately 30 minutes, and your responses will be anonymised.

Course Attendance

·       Did you attend the ENT Emergency Course?

·       Why did you not attend the course?

·       Did your local induction prepare you for clinical practice? Can you provide examples?

·       Have you attended any other ENT emergency course? Have you seen any other courses advertised?

ENT Placement

·       How did you feel prior to starting your ENT placement?

·       How would you rate your knowledge and practical skills prior to starting in ENT?

·       Did you feel sufficiently prepared for starting your ENT placement?

·       Have you ever considered a career in ENT?

·       Did your career aspirations influence your decision not to attend the course?

Course Structure

·       Who do you think should teach induction? Consultants or registrars?

·       How would you ideally like an ENT Induction course to be structured?

·       What topics would you like the session to cover? How do you think it should be structured?

·       What are your thoughts on the following course structures:

    - A full day of lectures, followed by a full day of practical skills

    - A half day of lectures, followed by a half day of practical skills

    - Pre-course lecture material followed by in-person practical skills

Cost

·       What would be the maximum amount of money you would be willing to spend on an induction course?

·       Do you think SHOs should pay for an induction to a speciality?

·       Do you have access to a study budget for courses?

Timing

·       Would you have to attend the course on a scheduled off day? Did this influence your decision not to attend the course?

·       How do you feel about participating in courses out of hours?

·       Are you granted time in lieu for courses? How easy is it to request study leave?

Inductions

·       What induction was provided by your local department?

·       What is your experience of induction in other specialities?

·       Was your local induction sufficient for commencing a placement in ENT?

Semi-Structured FGD 3: Registrar Faculty

Thank you for agreeing to take part in this study looking at the perceptions of ENT SHO induction. This session will last approximately 30 minutes, and your responses will be anonymised.

Course Attendance

·       Did you assist as faculty for the ENT Emergency Course? Why?

·       Have you previously assisted as faculty? Do you plan to help out in the future?

·       What motivates you to assist with the Emergency Course? How does the course benefit you? What are the disadvantages of participating as faculty?

·       What would you change about the course? Why?

·       Do you work with any SHOs who have attended the ENT SHO Emergency Course? Do you think it prepares them for clinical practice?

·       Do you think participation as faculty should be mandatory?

·       Have you assisted faculty with any other ENT emergency course? How does this course compare? Have you seen any other courses advertised?

Course Structure

·       Do you think the course should be taught by consultants or registrars?

·       What did you think of the current structure and timing of the course? (i.e., morning lectures, afternoon practical sessions)?

·       How would you ideally like the course to be structured?

·       Are there any additional topics or sessions you think the course should cover?

·       What was your opinion on the duration of the course?

·       How would you feel if we extended the course over two days? e.g., a full day of lectures and a full day of practical sessions

·       How would you feel if we shortened the course? e.g., pre-recorded morning lectures could be watched in students’ own time, and we could just provide the practical course. Do you think people would watch them?

Teaching

·       Did you receive any formal teacher training prior to helping as faculty?

·       Did you feel sufficiently prepared for delivering your training station?

Cost

·       Do you think the price of the course is fair?

·       What do you think the maximum course price should be?

·       What would be the maximum amount of money you would be willing to spend on an induction course?

·       Do you think SHOs should pay for an induction to a speciality?

·       Were you paid for your participation?

·       Would payment influence whether you assisted as faculty?

Timing

·       Did you have to attend the course on a scheduled off day? Did this influence your decision to attend the course?

·       How do you feel about participating in courses out of hours?

·       Were you granted time in lieu?

Inductions

·       What induction is provided by your local department?

·       Do you feel your local induction is sufficient for commencing a placement in ENT?

Semi-Structured FGD 4: Registrar Non-Faculty

Thank you for agreeing to take part in this study looking at the perceptions of ENT SHO induction. This session will last approximately 30 minutes, and your responses will be anonymised.

Course Attendance

·       Did you assist as faculty for the ENT Emergency Course? Why?

·       Have you previously assisted as faculty? Do you plan to help out in the future?

·       What motivates you to assist with the Emergency Course? How does the course benefit you? What are the disadvantages of participating as faculty?

·       If you have helped out before, do you know how the course is structured? What would you change about the course? Why?

·       Do you work with any SHOs who have attended the ENT SHO Emergency Course? Do you think it prepares them for clinical practice?

·       Do you think participation as faculty should be mandatory?

·       Have you assisted faculty with any other ENT emergency course? Have you seen any other courses advertised?

Course Structure

·       Do you think the course should be taught by consultants or registrars?

·       What did you think of the current structure and timing of the course? (i.e., Morning lectures, afternoon practical sessions)?

·       How would you ideally like the course to be structured?

·       Are there any additional topics or sessions you think the course should cover?

·       What was your opinion on the duration of the course?

·       How would you feel if we extended the course over two days? e.g., A full day of lectures and a full day of practical sessions

·       How would you feel if we shortened the course? e.g., Pre-recorded morning lectures could be watched in students’ own time, and we could just provide the practical course. Do you think people would watch them?

Cost

·       Do you think the price of the course is fair?

·       What do you think the maximum course price should be?

·       What would be the maximum amount of money you would be willing to spend on an induction course?

·       Do you think SHOs should pay for an induction to a speciality?

·       Would payment have influenced whether you assisted as faculty?

Timing

·       Did you have to attend the course on a scheduled off day? Did this influence your decision to attend the course?

·       How do you feel about participating in courses out of hours?

Inductions

·       What induction is provided by your local department?

·       Do you feel your local induction is sufficient for commencing a placement in ENT?

Semi-Structured FGD 5: Consultant

Thank you for agreeing to take part in this study looking at the perceptions of ENT SHO induction. This session will last approximately 30 minutes, and your responses will be anonymised.

Course Attendance

·       Did you assist as faculty for the ENT Emergency Course? Why?

·       Have you previously assisted as faculty? Do you plan to help out in the future?

·       What motivates you to assist with the Emergency Course? How does the course benefit you? What are the disadvantages of participating as faculty?

·       If you have helped out before, do you know how the course is structured? What would you change about the course? Why?

·       Do you work with any SHOs who have attended the ENT SHO Emergency Course? Do you think it prepares them for clinical practice?

·       Do you think participation as faculty should be mandatory?

·       Have you assisted faculty with any other ENT emergency course? Have you seen any other courses advertised?

Course Structure

·       Do you think the course should be taught by consultants or registrars?

·       What did you think of the current structure and timing of the course? (i.e., Morning lectures, afternoon practical sessions)?

·       How would you ideally like the course to be structured?

·       Are there any additional topics or sessions you think the course should cover?

·       What was your opinion on the duration of the course?

·       How would you feel if we extended the course over two days? e.g., A full day of lectures and a full day of practical sessions

·       How would you feel if we shortened the course? e.g., Pre-recorded morning lectures could be watched in students’ own time, and we could just provide the practical course. Do you think people would watch them?

Cost

·       Do you think the price of the course is fair?

·       What do you think the maximum course price should be?

·       What would be the maximum amount of money you would be willing to spend on an induction course?

·       Do you think SHOs should pay for an induction to a speciality?

·       Would payment have influenced whether you assisted as faculty?

Timing

·       Did you have to attend the course on a scheduled off day? Did this influence your decision to attend the course?

·       How do you feel about participating in courses out of hours?

Inductions

·       What induction is provided by your local department?

·       Do you feel your local induction is sufficient for commencing a placement in ENT?

Appendix B

**Table 6:** Coded reflexive thematic analysis from direct quotes

Theme	Subtheme	Code	Examples
1: Pedagogical Framework	1.1: Lectures	1.1.1: Content	CONS 1: “I think you need those lectures to cover the basic sort of ENT conditions”
			REG 2: “We took out dizziness. I think that works well…. We added in ‘post-op complications’ which are far more relevant”
			REG 1: “What would be interesting to know is actually how much of [the information] is being retained”
			SHO 1: “There were quite a few tables which delineated specific ways to manage certain scenarios… That kind of stuff you don’t really find in textbooks and it’s not something you come across without experience”
			SHO 3: “I don't think purely knowledge-based content is sufficient for induction”
		1.1.2: Structure	SHO 1: “You get a bit of information overload”
			SHO 2: “Maybe [provide] something for people to look at prior to the day itself, so they can get familiar”
			SHO 1: “I enjoyed it a lot and found it really useful”
			SHO 2: “If you were trying to do the practical [sessions] in between each relevant tutorial… that would be difficult logistically”
			SHO 1: “It was useful that I had least heard about some of the skills that we were coming across in the afternoon and… the equipment”
			REG 1: “I think it’s really difficult to get that much content across to people and keep them engaged”
		1.1.3: Handouts	SHO 1: “Maybe handouts would be helpful, so people can scribble notes”
			REG 2: “I probably wouldn’t look at them again”
			REG 2: “They get the book, so they get the information in a slightly different format”
	1.2: Experiential Learning	1.2.1: Proformas	REG 4: “It'd be nice to have like a structure of points that you ideally should cover… To make sure you don’t forget anything”
			REG 4: “It would be really good if we have new trainees… so they know what they need to cover”
			REG 5: “We often run out of equipment and I forget what’s supposed to be in each box… I think it would be useful to just have a list”
			REG 5: “Having some reference pictures would be helpful”
		1.2.2: Structure	SHO 5: “I think a good way to structure the course would be… station-based. So the consultant and the registrar demonstrate the station themselves first, and then you have a go after”
			SHO 1: “The practical procedures sort of wake you up as it involves a different kind of thinking… it’s something to look forward to”
			REG 5: “I think incorporating a case-based element to the practical sessions [would be] a really good idea”
			REG 5: “[SHOs] want to talk through the anatomy as well as performing the procedure”
			CONS 1: “The practical stuff people love… but it’s really helpful”
			REG 1: “Those are the key [practical skills]… you know if your SHO can do those things on call, you’re super happy as a registrar”
		1.2.3: Evaluation of Existing Practical Sessions	SHO 2: “My favourite stations were nasoendoscopy and learning to drain quinsies. I found those really valuable”
			REG 5: “The [quinsy] models we use are a bit fake and it’s easy to aim for the cod liver oil capsule”
			REG 5: “It is great practicing [flexible nasendoscopy] on each other”
			REG 6: “Taking something out of the ear is really simple. It’s mostly about your communication… And that’s something you can only really learn on the job, it’s super hard to simulate”
			REG 2: “I wonder whether anterior and posterior [epistaxis] could be combined? Because they are very similar… We could put in that dual lumen pack, which is way easier… Most trusts have them now”
			REG 5: “I think the surgical airway station is not something that’s really at SHO level… I guess it’s just useful for anatomical teaching really”
			CONS 1: “The only airway issue I can think of from an SHO perspective is a post-thyroid haematoma… I think that could probably just be covered as a lecture”
			REG 1: “I think introducing the cricothyroidotomy trainer is good”
			Foreign Bodies:
		1.2.4: Recommendations for Additional Practical Sessions	REG 4: “I’d really like to add some extra stuff into [the microscopy] session, [like] removing foreign bodies”
			REG 1: “I think it would be good to teach them how to swaddle a child and proper positioning”
			Suturing:
			REG 6: “Maybe a station on suturing”
			REG 1: “We all came out of medschool being able to suture because we all established our surgical interest from quite a young stage… but that's not as common these days”
			Manipulation of Nasal Fractures (MUA):
			REG 1: “I guess MUA isn’t that time-sensitive. It tends to be more in an emergency clinic scenario. And then you have time to go and grab your reg”
			REG 1: “The communication around it is far more important. The practical procedure doesn’t require much dexterity”.
			REG 1: “It's the decision-making process of whether it even needs doing that is tricky”
		1.2.5: Formative Assessment	SHO 5: “A mark scheme [would help you] to work out… what you needed to improve on”
			SHO 4: “I wouldn’t like it… I would feel like I was being judged”
	1.3: Flipped Classroom	1.3.1: Positive Perspectives on Flipped Classrooms	REG 1: “If you have pre-recorded lectures, you’ve got the chance to do it perfectly. So you're going to give the most optimum version of that lecture”
			REG 1: “You would have even more time for the practical stuff”
			REG 1: “Instead of [the SHOs] taking a full day out of a weekend, they could then use a half day of CPD or admin time to catch up”
			REG 1: “It would also broaden the accessibility… we could send it to people who weren’t able to attend… it would be free of charge”
			REG 1: “It would give the opportunity to revisit material [from the course]”
			REG 1: “That would enable us to double the number of SHOs we can help”
			SHO 6: “I think it works, but it really requires the person to be engaged”
		1.3.2: Negative Perspectives on Flipped Classrooms	REG 5: “[Lectures are] good to generate discussion”
			CONS 1: “Once people start asking questions… [it] opens like a whole new explanation topic, which you'll then miss out online”
			CONS 1: “A lot of people won't look at it or will try and skim through it really quickly… they just won’t engage”
			SHO 5: “If the pre-recorded lectures got too long, I’d probably just Google it… I would find the shortest video possible to get the information across quicker”
			SHO 6: “I’m not sure I would do it properly. And then I’d miss out as I wouldn’t get the practical stuff as much”
			SHO 6: “I would already be giving up a Saturday to attend the course, so then that’s like even more time out [to review pre-recorded material]”
			SHO 2: “I’m always going to choose being there in person on the day. I prefer the social interaction”
			SHO 2: “You make it quite funny when you teach it but I’m not sure how well that would translate into a video”
	1.4: Comparative Educational Analysis	1.4.1: Local Induction	CONS 1: “Trusts wouldn't have access to our models”
			CONS 1: “You've got to cover all the other things, like how to take annual leave… who your supervisors are, how the rotas work… So that then means that you end up having less time to cover the ENT stuff”
			CONS 1: “It involves lots of lectures”
			REG 2: “We don’t really get any opportunity to do any practical [skills]”
			REG 5: “I think there’s more of a cultural change into doing stuff locally and trying to support the SHOs better”
			REG 1: “[it’s] kind of didactic… And it has none of the practical sessions”
			SHO 3: “What you really need is that hands on shadowing experience… And there’s no way of making that better other than jumping in at the deep end”
		1.4.2: Other ENT Emergency Courses	REG 4: “The West Midlands one is definitely much more comprehensive”
			REG 4: “Z Course… it’s just an afternoon of practical sessions, which is good but I also think having the lectures in the morning just gives everything a bit more of a context”
			REG 2: “I’ve also heard of these airway simulation courses which include anaesthetists, surgeons, and a scrub nurse… I think that's probably beyond the scope of our current course”
			SHO 2: “I've seen [an airway management course], but I feel like it was more geared towards [registrars]”
		1.4.3: Other Specialty Inductions	SHO 5: “You get departmental teaching as you go through your rotation… But that’s pretty useless if you are only hearing about important stuff at the end of your rotation”
			SHO 6: “The other inductions I’ve had have been way less hands-on”
			SHO 5: “We’ve had [an induction] where it’s like ‘here’s the staffroom, here’s the kitchen’. But nothing that actually aims to give you knowledge”
2. Facilitators and Barriers to Participation	2.1: Facilitators for Faculty Participation	2.1.1: Helping Others	CONS 1: “We want to give up our time to teach others”
			CONS 1: “You get SHOs [to] start the job feeling more confident and usually… more competent”
			CONS 1: “I personally went on the course and I found it really helpful. So I think it's useful then to help the next generation”
			CONS 1: “I think it doesn't need to directly benefit you in terms of your career”
			REG 2: “I started off doing it because it benefits others”
		2.1.2: Enjoyment of Teaching	REG 4: “I also just enjoy teaching”
			REG 3: “It's good to share knowledge and see how knowledge is shared”
		2.1.3: Enhancing SHOs’ Capabilities to Support ENT Registrars During On Calls	CONS 1: “You hope that some of your SHOs will come on the course… the more confident and competent your SHOs are, the less you [will] get called out of hours”.
			REG 1: “It's in all of our interests for especially local SHOs to be trained well”
		2.1.4: Simplifying the Training of New SHOs	CONS 1: “It means you end up having to not teach quite so long at the beginning”
			REG 1: “I’ve found [local induction doesn’t] have the time or resources to do it properly. So from a…service needs point of view, I think it’s good”
			REG 2: “It benefits the SHOs so that they can… get to grips with ENT before they come onto the ward”
		2.1.5: Impact on SHO Performance	CONS 1: “I find those who have been on the course tend to be… one step ahead of those who haven't”
			CONS 1: “The course teaches a wide variety of skills, but I think the confidence extends further to other skills that aren't taught on the course as well… [for example] a fractured nose and manipulation under anaesthetic”
			CONS 1: “Those who haven't been on the course are much more hesitant to try [new skills]”
			CONS 1: “They [do] some clinical skills which they wouldn’t have otherwise practiced”
			REG 4: “It just seems like [SHOs who attend] ask more relevant questions”
			REG 4: “They can build upon that confidence when they come to spend more time in the department”
			REG 4: “It just gives them a bit of a structure to relate back to”
		2.1.6: Teacher Training Opportunities	REG 4:”It’s important to keep refreshing your teaching skills”
			REG 2: “The curriculum states you need an observation of teaching, and the course can give you that”
			CONS 1: “I think for those maybe who haven't done any teaching at all, then potentially it’s beneficial”
		2.1.7: Payment of Facilitators	REG 4: “Whether I get paid or not wouldn't really affect it… I just enjoy teaching on the course, and I'm happy to help”
			REG 3: “I was not expecting to get paid”
			REG 1: “If you’re just coming for the afternoon…it's not that much money”
			REG 1: “[Paying the faculty] would mean increasing the cost to the SHOs”
			REG 1: “From a faculty perspective it would be super annoying, because you’re then going to have to have people employed… and then you have to do HR checks… and then it's going to get taxed. I think that's actually going to make life more difficult”
		2.1.8: Networking Opportunities	REG 2: “I quite enjoyed the sort of social side of things”
			REG 3: “I’m new to the country so I'm trying to get integrated into the system”
			CONS 1: “Certainly, when I have offered or not offered [to help on the course], it's never crossed my mind what will the bosses think”
			CONS 1: “I'm sure… there are lots of people if they were told, oh, the bosses will know whether you've participated, that would influence their decision”
		2.1.9: Additional Benefits	CONS 1: “I think it’s very reasonable to say… those who are teaching… should get some sort of compensation, whether that be a day back in lieu, whether it be a meal out, whether it’s a gift”
			REG 1: “There's the dinner which is good. But you know, I probably think my time is worth more than a free dinner.”
			REG 5: “We pay for people’s meal out afterwards. And that’s a really nice gesture. I’m not sure people are always keen to stay on after the course as that’s more hours out of their day”
			REG 2: “We give formal [station-specific] feedback, it’s not just the certificate”
		2.1.10: Mandatory Attendance for Registrars	REG 5: “I think it’s difficult to mandate it when it’s on a weekend”
			REG 3: “I think it’s really important to develop registrars in being adept at teaching and training as well as supervising. It’s a good course to develop that skill”
	2.2: Barriers for Faculty Participation	2.2.1: Personal Reasons	CONS 1: “I was on maternity leave”
			REG 1: “I was meant to have plans”
		2.2.2: Teacher Training Opportunities	REG 1: “Most of us have done enough [teaching sessions], and doing another… is not quantifiably important”
		2.2.3: Lack of Direct Benefit	CONS 1: “There’s no guarantee that your SHOs will attend that course… So there’s no guarantee by teaching on that course that you’ll get that direct benefit”
			REG 1: “It’s disappointing if the majority of trainees are not from this region or may not have an upcoming ENT rotation. But it doesn’t negatively influence my willingness to participate”
		2.2.4: Course Timing	CONS 1: “It’s the weekend. I think that's the main reason why people don't give up their time”
			REG 1: “My motivation would be higher if I was taking a day off work”
			REG 1: “If it actively detracts from something I have planned in my personal life, then I'm afraid that's not for me”
			REG 1: “On call rotas are really variable, so coming in on a weekend will feel different to different people”
	2.3: Facilitators for SHO Participation	2.3.1: Acquiring ENT Knowledge	CONS 1: “[The SHOs] are responsible for making sure [they’re] not only competent and [they’ve] got the knowledge, but also [they’re] performing the best they can”
			CONS 1: “They also get a handbook”
			CONS 1: “There’s not currently enough time for just learning on the job”
			“REG 1: I think it's a good course that gives them a lot more time compared with local inductions”
		2.3.2: Development of ENT Practical Skills	CONS 1: “They [get] a bit of practical experience, which they haven’t necessarily had at medical school or other specialties”
			SHO 1: “The practical elements… was the core part of what I wanted to achieve… to get some exposure to the key skills”
		2.3.3: Career Development	SHO 2: “It would really help me with getting a [future job] in ENT”
			SHO 2: “These courses count towards my core [surgical] training application”
			SHO 2: “It just further confirmed that I definitely have an interest in ENT”
			SHO 1: “I’m leaning more towards Orthopaedics, but I still think it’s helpful… experience and exposure”
			SHO 5: “These days, people are strongly motivated by portfolio points. So if the course would give you some kind of points you could put on your CV, I think that would really get people through the door”
			REG 6: “A really important part… is the social component. It’s a good networking opportunity”
			REG 6: “One of the other aims of the course is to get people enthusiastic about ENT… that’s equally as important as teaching”
			REG 6: “I've had two people approach me about writing [research] papers”
			CONS 1: “If you feel like you've got that confidence, I think it makes you enjoy the job. And if you enjoy the job, you then become interested [in pursuing a career in ENT]”
		2.3.4: Impact on SHO Confidence	SHO 1: “The course gives you the confidence to start managing [ENT Emergencies], and know when to escalate up to the registrar”
			SHO 1: “I could follow the structure we spoke about on the course”
		2.3.5: Mandatory Attendance for SHOs	REG 5: “I think it’s difficult to mandate it when it’s on a weekend”
			CONS 1: “I really think the course should almost be compulsory”
			REG 1: “Yeah, if you get [the local department] to pay for it”
			REG 1: “What do you do for the ones that don’t do it? Are you going to say, well then they don’t get any induction?”
	2.4: Barriers for SHO Participation	2.4.1: Personal Reasons	SHO 4 and 5: “I just didn’t hear about it”
		2.4.2: Changing Attitudes to Training	CONS 1: “Back in my day, you wanted to be good at your job… You would stay late so that you would learn… Now I think this has shifted to just a job which pays the bills”
			CONS 1: “More people now want to work the hours that they are paid for… Whereas more senior trainees have more of a sense of responsibility for making sure that we're keeping up to date with our own learning”
			CONS 1: “Morale's rubbish. When morale's low, people don't… put in the extra hours that they would have previously”
			REG 1: “This generation of doctors are generally unsatisfied… They're saying ‘I feel unsupported’… When you then say, here is your training, they don’t seem to be happy with that”
		2.4.3: Course Timing	REG 1: “You’re basically asking them to give up a day of their weekend”
			SHO 5: “I probably wouldn't change my plans for the course”
			SHO 2: “I think that would have deterred me if I had to request leave or organise a rota swap. Especially so soon into starting a rotation”
		2.4.4: Clinical Commitments	CONS 1: “I’ve led multiple inductions and inevitably one of the SHOs is on call or on nights so they haven't been able to attend”
			CONS 1: “There's just no provision for covering their on call so that they can attend”
		2.4.5: Adequacy of Local Training	CONS 1: “I think if the trust could provide [a better induction] than they currently do, then I would say that [attending the WMEEC] could be optional”
			SHO 5: “I think my knowledge isn't that bad, because I can read my medical school notes. And they pretty much covered most of the stuff I’ve come across so far”
		2.4.6: Cost	REG 5: “I think quite a few people are motivated to attend of their own accord, regardless of whether they have to pay or not”
				3: Operational Structure	3.1: Temporal Structure	3.1.1: Attending Courses at Weekends	CONS 1: “It only runs three times a year, so it’s not that big a deal… you don’t have to do it every single time”
REG 1: “[Attendees] are often more grateful… they recognise you’re doing it in your unpaid weekend time”						
REG 4: “I think it’s good for me to be on an off day, because you don’t have to rearrange clinic, you’re not missing theatre… there is less to organize”						
REG 4: “I’m less likely to be on call”						
REG 4: “A lot of people just come for the afternoon…You still have the morning and evening free”						
SHO 4: “It wouldn’t be my first choice”						
SHO 3: “It wouldn’t deter me. I’d be willing to go on any ENT course… But someone who doesn’t want to do ENT might not do the same”						
3.1.2: Attending Courses on Normal Working Days	REG 1: “It’s quite easy to get the time off work… because they get planned really far in advance”				
	REG 1: “It would depend slightly on what I had on that day. If it was a really good operating list, my desire to go and volunteer on a course might be less than helping on a weekend”				
	REG 1: “You know [SHOs] won’t be able to get study leave in work time so soon into a new post”				
	SHO 2: “I think that would have deterred me if I had to request leave… or organise a rota swap”				
	SHO 1: “The department should facilitate the time to be taken [for this course]”				
3.1.3: Days in Lieu	CONS 1: “I think it’s very reasonable to say… those who are teaching on it should get… a day back in lieu”				
	CONS 1: “I know attitudes are changing. I think a lot of people now wouldn’t be willing to give up a Saturday… without a day back”				
	REG 1: “The day in lieu probably wouldn't make much of a difference to me”				
	REG 1: “If you could…get a half day back in lieu, I think you'd have great uptake”				
	REG 2: “It would be difficult…You'd need all the trusts on board”				
	REG 5: “I'm not sure people… would be that interested in it. Because I think that Saturday is a precious time when, in general, more of your family and friends are available. And having a random off day during the week isn't necessarily beneficial”				
	SHO 1: “If you can’t get that same level of education locally, I think you should get time back”				
	SHO 2: “I didn’t get that time back. I think they were concerned that it then takes time away from your normal working schedule”				
3.1.4: Clinical Commitments	SHO 3: “I’ve always been on nights and always ended up missing the local induction… I felt very much left in the dark… It felt chaotic… I found that it affected people’s overall perception of me as it looked like I didn’t care”				
	REG 2: “Having one designated time to give an induction to everyone is really difficult… You've always got someone on nights. You've always got someone that's on call etc”				
	REG 5: “I think the local departments will say it’s not their responsibility to ensure people are trained up, or staffing cover is provided”				
3.2: Temporal Scope	3.2.1: One-Day Course	REG 1: “I think the current format is good”		
		SHO 2: “If it’s all just in one day… then you can relax for the rest of the weekend”		
		REG 5: “The lunch session could be shorter. They only really need half an hour”		
	3.2.2: Two-Day Course	SHO 1: “I find it difficult to concentrate… [if the course was] spread out across two days it would mean we could take it at a slower pace”		
		SHO 1: “If you were on call, you would have to switch two days instead of one, which might be harder”		
		REG 2: “I think two days would be too much”		
		REG 1: “They wouldn’t get a day off before starting work on the Monday”		
		REG 1: “If people are coming from elsewhere, then they have to start thinking about accommodation”		
	3.2.3: Half-Day Course	REG 2: “It doesn’t need shortening”		
		REG 1: “If people are travelling from afar, are they going to come all that way for half a day?”		
3.3: Financial Considerations	3.3.1: Perspectives on Current Course Costs	CONS 1: “I think £65 is a very good deal for an all-day course”		
		CONS 1: “When I attended the course potentially 10 years ago now, it was very much seen as not only is this good value, it's a good opportunity”		
		REG 1: “That constitutes excellent value for money”		
		SHO 5: “I’m not interested in becoming a surgeon so I wouldn’t want to spend a lot of money on a really basic surgical course”		
		SHO 4: “I’m an F1…I’ve got a lot of debt, I don’t have any money. So I would be happy to go on the course, but I can’t even afford to go on a course like that, even if I’d get the money back with study budget”		
		REG 2: “We’re not here to make a profit and the basic equipment has already been purchased”		
		SHO 2: “It felt almost subsidized, just because of the food and equipment”		
		SHO 2: “I’d only pay more if I know with certainty I would get it back”		
	3.3.2: Study Budget	CONS 1: “I think it should come out of study budget, because it's helping you do your job, and we are meant to be trainees”		
		SHO 3: “It’s easy to claim the money back”		
		SHO 2: “It’s such a headache often I don’t bother claiming the money back”		
		SHO 1: “The reimbursement is not prompt”		
	3.3.3: Self-Funding of Courses	CONS 1: “If you weren't a trainee, then you could argue maybe people should pay”		
		CONS 1: “I think a lot of people now wouldn’t be willing to give up a Saturday… without [getting] the money back”		
		CONS 1: “The trust should be paying for it”		
		REG 5: “I think quite a few people are motivated to attend of their own accord, regardless of whether they have to pay or not”		
		SHO 5: “I wouldn’t mind paying”		
		SHO 2: “I paid for it and I was happy”		
		SHO 1: “It should be free of cost”		
		REG 1: “I don't think they should be paying out of their own pocket without getting that reimbursed”		
		SHO 3: “I think there is an element of responsibility for the local department to ensure the new doctors are appropriately trained. And that includes sponsoring them to go on extra courses, if they are not able to provide the same level of teaching”		
		SHO 2: “For those who have a job in ENT, it should be totally paid for….But for those who are just generally interested in ENT… then you could justify for the SHO to pay for it”		
3.4: Participant Demographics	3.4.1: Prioritising Local SHOs	CONS 1: “I think priority should be given to West Midlands trainees”		
		CONS 1: “[Non-local SHOs] might feel less embarrassed to ask questions because there's no one to judge them… [they think] ‘I'm never going to meet these people again’”		
		REG 1: “I think it's great that we open it up to [non-local SHOs], as long as you've got the spaces”		
	3.4.2: Allied Healthcare Practitioners	CONS 1: “I think ENT SHOs should be prioritized, because they’re the ones who are going to be doing the on-call rota and seeing emergencies”		
		CONS 1: “If there are spaces on the course, I think it's very reasonable for other allied health professionals… who are going to see ENT conditions, like advanced nurse practitioners or those working in A+E”		
3.5: Instructional Facilitators	3.5.1: SHOs	SHO 5: “I feel like SHOs wouldn’t really know more than me”		
		SHO 4: “I think SHOs will have the most recent practical skill-based knowledge”		
	3.5.2: Registrars	CONS 1: “I think you can be more open and honest when you have somebody sort of similar grade to you”		
		CONS 1: “There might still be that slight [hierarchy], but I think you need that because you need somebody who's got more knowledge than you”		
		SHO 2: “They just are way more approachable”		
		SHO 6: “I find registrars more enthusiastic”		
		REG 4: “I also think as we have only just recently stopped being SHOs, we have more of an understanding of what they might find difficult”		
		REG 3: “Chances are when you're the SHO on call, you’re mostly going to be liaising with registrars. So it’s more important to develop that sort of interaction”		
	3.5.3: Consultants	CONS 1: “I think a lot of consultants don't really know what's seen in an emergency clinic or when on call”		
		REG 5: “I think getting someone who has done [basic ENT practical skills] more recently would be way more helpful”		
		SHO 6: “Consultants can be quite intimidating”		
		SHO 5: “I think maybe for lectures consultants would be fine as that’s less interactive and they probably have more knowledge”		
	3.5.4: Formal Teacher Training	REG 1: “The course doesn’t provide any formal teacher training”		
		REG 3: “Most of the knowledge I imparted was based on my clinical experience… I’ve taught before so don’t really feel I needed any help”		
		REG 5: “I was paired up in the afternoon with more senior registrars… then I got to know what other people were teaching… you could share ideas”		
